# Silica Treatments: A Fire Retardant Strategy for Hemp Fabric/Epoxy Composites

**DOI:** 10.3390/polym8080313

**Published:** 2016-08-22

**Authors:** Francesco Branda, Giulio Malucelli, Massimo Durante, Alessandro Piccolo, Pierluigi Mazzei, Aniello Costantini, Brigida Silvestri, Miriam Pennetta, Aurelio Bifulco

**Affiliations:** 1 Department of Chemical Materials and Industrial Production Engineering (DICMaPI), University of Naples Federico II, P.le Tecchio 80, Naples 80125, Italy; massimo.durante@unina.it (M.D.); anicosta@unina.it (A.C.); brigida.silvestri@unina.it (B.S.); miriam.pennetta@gmail.com (M.P.); aur.bifulco@libero.it (A.B.); 2 Department of Applied Science and Technology, Politecnico di Torino, Viale Teresa Michel 5, Alessandria 15121, Italy; giulio.malucelli@polito.it; 3 Centro Interdipartimentale di Ricerca sulla Risonanza Magnetica Nucleare per l’Ambiente, l’Agroalimentare e di Nuovi Materiali (CERMANU), Via Università 100, Naples 80055, Italy; alessandro.piccolo@unina.it (A.P.); pierluigi.mazzei@unina.it (P.M.)

**Keywords:** hemp/epoxy composites, waterglass treatments, solid-state NMR, flame retardancy, cone calorimetry tests, three-point bending tests, vacuum bag molding

## Abstract

In this paper, for the first time, inexpensive waterglass solutions are exploited as a new, simple and ecofriendly chemical approach for promoting the formation of a silica-based coating on hemp fabrics, able to act as a thermal shield and to protect the latter from heat sources. Fourier Transform Infrared (FTIR) and solid-state Nuclear Magnetic Resonance (NMR) analysis confirm the formation of –C–O–Si– covalent bonds between the coating and the cellulosic substrate. The proposed waterglass treatment, which is resistant to washing, seems to be very effective for improving the fire behavior of hemp fabric/epoxy composites, also in combination with ammonium polyphosphate. In particular, the exploitation of hemp surface treatment and Ammonium Polyphosphate (APP) addition to epoxy favors a remarkable decrease of the Heat Release Rate (HRR), Total Heat Release (THR), Total Smoke Release (TSR) and Specific Extinction Area (SEA) (respectively by 83%, 35%, 45% and 44%) as compared to untreated hemp/epoxy composites, favoring the formation of a very stable char, as also assessed by Thermogravimetric Analysis (TGA). Because of the low interfacial adhesion between the fabrics and the epoxy matrix, the obtained composites show low strength and stiffness; however, the energy absorbed by the material is higher when using treated hemp. The presence of APP in the epoxy matrix does not affect the mechanical behavior of the composites.

## 1. Introduction

In the last ten years, the interest in natural fiber-reinforced polymer composites exhibited a significant growth as far as fundamental research and their industrial applications are considered: indeed, these materials are not expensive, fully or partially recyclable and also biodegradable [1,2,3,4]. Apart from wood, such plants as flax, jute, sisal, kenaf, cotton, hemp, bamboo, banana, pineapple, ramie, etc., have been utilized as a source of lignocellulosic fibers and very often exploited as the reinforcement of composites. Their convenience, renewability, low density and price, as well as acceptable mechanical features make them very attractive “green” alternatives to glass, carbon and man-made fibers, commonly employed for the manufacturing of composites [5,6,7].

As compared to synthetic fiber-reinforced composites, the natural fiber-containing counterparts are more environmentally friendly, hence deserving use in a wide range of applications, including transportation (cars, railway coaches, aerospace vehicles), military purposes, building and construction industries (partition boards, ceiling paneling), packaging, consumer products, etc. Among the different natural fibers, despite its current limited availability, hemp is particularly raising interest for green composite manufacturing, as it is an inexpensive renewable resource and shows low density, high specific strength with respect to glass or aramid fibers and good biodegradability, as well [8,9].

Until the early 1900s, industrial hemp was a valuable crop used all over the world for its strong fibers and oil seeds. Indeed, the Yearbook of the United States Department of Agriculture documents well the significant spread of hemp cultivation all over the world in 1913 [10]. By the 1930s, the commercial cultivation of hemp began to decrease, due to its reduced demand compared to increasingly popular synthetic fibers. Nowadays, hemp is regaining interest for green composite manufacturing, thanks to the ecological and economic advantages over conventional composites. 

One of the major drawbacks of the aforementioned composites, including those containing hemp, is their ease of flammability when exposed to a heat flux or a flame source [11]: this behavior can significantly restrict the application fields of these materials, especially when the possibility of the use of the composites is strictly related to specific regulatory fire tests that have to be passed, hence ensuring public safety.

The natural fiber-reinforced composites, when exposed to fire or any other high-intensity heat source, undergo thermal decomposition and combustion processes according to the adopted experimental conditions. In particular, the time to ignition of the composites and the intensity of combustion process are significantly affected by such parameters as heat and mass transfer from and to the composite material, oxygen concentration and the circulation of gas in the area where combustion takes place. Important issues comprise the rate of flame spread, Heat Release Rate (HRR), mass loss and carbonization rates. The ease of the combustibility of a composite material depends on a number of factors, including the nature of the composite and its components (i.e., type of natural fibers and polymer matrices), its density, structure, thermal conductivity, humidity content, and so on.

The fire retardancy of composite materials can be enhanced exploiting different strategies. First, it is possible to add different kinds of fire retardants in liquid or solid form during the manufacturing process: as a result, they are embedded within the composite structure. More specifically [12,13,14]:

(1) the natural fibers can be impregnated with a solution of the flame retardant;

(2) the flame retardant can be incorporated into the adhesive system (e.g., melamine urea formaldehyde condensate, pea protein);

(3) the fibers can be subjected to a surface treatment;

(4) the fibers can be mixed with the flame retardant before the addition of an adhesive.

In these conditions, the performed treatments should exhibit an acceptable thermal stability that prevents the activation of the flame retardant during the fabrication step of the composite material. 

Second, it is possible to apply fire retardant finishing treatments at the end of the fabrication process: this is very often performed when intumescent flame retardant coatings are exploited [15].

The treatment with non-combustible components provides natural fibers or lignocellulosic particles with a fire retardant coating: in these conditions, fire retardants tend to migrate inside the flammable materials, thus resulting in the fire protection of the latter.

The different flame retardant additives (5–10 wt % in relation to dry mass) used for lignocellulosic materials comprise ammonium salts of phosphoric acid (i.e., melamine phosphate, ammonium polyphosphate), zinc chloride, boric acid, salts of sulfuric acid, zinc borate, vermiculite, aluminum hydroxide, magnesium hydroxide, expandable graphite and pentaerythritol (as the carbon source for intumescent systems) [12,13,16,17,18].

Unlike thermoplastic matrix-natural fiber composites, for which the open scientific literature reports several nice examples [19,20,21,22], the investigation of the fire behavior of thermosets reinforced with natural fibers has been only partially reviewed.

Manfredi et al. [23] investigated the thermal and fire behavior of composites based on unsaturated polyester or modified acrylic resins and reinforced with different natural fibers (namely, jute, flax, sisal) at 30 vol %. It was found that both the polymer matrices showed similar fire behavior, notwithstanding that modified acrylic resin-based composites gave rise to lower smoke as compared to unsaturated polyester-based counterparts, thanks to the char-forming properties of the former. Among the composites with modified acrylic resins, sisal-reinforced materials showed the highest fire risk and the worst fire behavior. Furthermore, jute fiber-containing composites exhibited quick growing, but short-lasting fire and, conversely, flax fiber-containing composites developed long duration, but slow growing fire.

Hapuarachchi and co-workers [24] assessed the potential application of industrial hemp fiber-reinforced sheet molding compound materials suitable for building applications, with particular emphasis on their fire behavior. The composites were added with aluminum trihydrate and subjected to cone calorimetry tests performed at 25 and 50 kW/m^2^. A significant decrease of the peak of the heat release rate was found for the systems containing aluminum trihydrate: this finding demonstrated that the obtained composites can compete with current building materials in terms of fire behavior.

Chai et al. [25] compared the flax-fiber-reinforced epoxy samples to glass-fiber-reinforced counterparts. It was found that glass fibers were able to provide partial protection from ignition, acting as a heat sink, as well as a physical barrier to the heat source; conversely, flax fibers were themselves susceptible to ignition and combustion with a higher peak of heat release rate compared to their glass fiber equivalents. In addition, the glass/flax hybrid laminates showed fairly good fire resistance with respect to flax-reinforced laminates. Their structural integrity and the resistance to fire was found to decrease with increasing the amount of natural reinforcement [26].

Very recently, Szolnoki et al. [9] prepared flame-retarded natural hemp fabric-reinforced epoxy resin composites. For this purpose, the hemp fabrics were treated according to three different methods: (i) immersion of preheated fabric into cold phosphoric acid solution (allowing the penetration into the capillaries of the fibers) and subsequent neutralization; (ii) reactive modification with an aminosilane-type coupling agent; (iii) the combination of the sol-gel surface coating with the first approach. The presence of phosphorus in the hemp fabrics turned out to decrease the flammability not only of the reinforcement, but also of the epoxy composites made thereof. In addition, it was possible to achieve V-0 classification (it means that burning stops within 10 s on a vertical specimen; drips of particles allowed as long as they are not inflamed) according to UL-94 standard (UL is the abbreviation for the Underwriters Laboratories, an independent organization in the United States to control and certificate product safety; furthermore, UL-94 is a flammability test performed on a defined specimen) rating by applying an amine-type phosphorus-containing curing agent in combination with the treated hemp fabrics.

In this paper, we prepared hemp fabric/epoxy composites by using vacuum bag molding. This technique has minimal impact on tool cost and imposes no limits on the part size process for producing large-scale composites; thus, the reduction in laminate flaws to improve part quality can be obtained at a competitive cost.

In particular, a new, inexpensive, simple and ecofriendly chemical strategy is exploited to coat hemp fabrics with a silica-based fire retardant layer, obtained by using a waterglass coating prepared in acidic conditions. In parallel, Ammonium Polyphosphate (APP), a very well-known flame retardant, is added to epoxy resin, aiming at assessing the possible joint effects of the flame retardant with the waterglass treatment. Very recently, this additive was used by our group [8] for conferring fire retardant properties to hemp/epoxy composites manufactured by the infusion process: the obtained results showed that the epoxy resin added with 16.32 wt % APP can be used in infusion processes of biocomposites without any impact on the technological feasibility of the process itself and provides significant enhancement as far as the fire behavior is considered.

Pursuing this research, in this work, we investigate the effect on the concurrent presence of surface-treated hemp fabrics and APP on the fire behavior of the obtained composites, assessed through cone calorimetry tests. The effect of the modification of hemp fabrics with the waterglass treatment is assessed through Scanning Electron Microscope (SEM), Fourier Transform Infrared (FTIR) spectroscopy and solid-state Nuclear Magnetic Resonance (NMR). Furthermore, Thermogravimetric Analysis (TGA) performed in nitrogen are exploited for assessing the thermal stability of both the waterglass-treated hemp fabrics and of their epoxy composites.

## 2. Materials and Methods

### 2.1. Materials

Sodium metasilicate and hydrochloric acid (37% ACS) reagents were purchased from Sigma Aldrich (St. Louis, MO, USA). Plain weave hemp fabrics (grammage: 160 g/m^2^, supplied by MAEKO S.r.l., Milan, Italy) and a two-component epoxy resin system (SX10 by MATES S.r.l., Milan, Italy) were used for fabricating composite laminates. The two components are reported by the supplier to be modified bisphenol A resin and modified cycloaliphatic polyamines. The epoxy resin was added with APP particles (white free-flowing powder) supplied by Tecnosintesi S.p.A. (Bergamo, Italy).

### 2.2. Methods

#### 2.2.1. Hemp Fabric Coating

A waterglass solution 0.1 M was acidified to pH = 2.5. Hemp fabrics were repeatedly soaked into this solution for 10 min and dried in an electric oven at 80 °C for 20 min. The treatment was repeated five times.

#### 2.2.2. Manufacturing of Hemp Fabric/Epoxy Composites

The composite laminates were fabricated by hand lay-up, positioning, on the mold, six layers of plain weave hemp fabric impregnated by the two-component epoxy resin system. The hand lay-up process was adopted in order to avoid the filtration of the filler in the resin by the reinforcement layers.

After impregnation, in order to increase the volumetric percent of reinforcement and to eliminate entrapped air, the laminates were enclosed in a vacuum bag, and polymerization was accomplished through a 48-h cure cycle at room temperature. The 3 mm-thick laminate contains 25 wt % of reinforcing agent; the 22 vol % was obtained as the ratio of the hemp fiber volume (estimated as the ratio of fiber weight and density, 1.4 g/cm^3^) to the laminate volume. Four different composites were produced by infiltrating hemp fabric sheets or treated hemp fabric sheets with epoxy or APP-charged epoxy. The obtained samples are coded as reported in Table 1.

### 2.3. Characterization Techniques

#### 2.3.1. FTIR Spectroscopy Measurements

FTIR transmittance spectra were recorded with a Nikolet 5700 FTIR spectrometer (Thermo Fisher, Waltham, MA, USA) using a single reflection Attenuated Total Reflectance (ATR) accessory with a resolution of 4 cm^−1^ and 32 scans and Thermo Scientific™ OMNIC™ Software Suite (v7.2, Thermo Fisher, Waltham, MA, USA, 2005).

#### 2.3.2. SEM-EDS-OM Microscopy

SEM images of samples were obtained on a Leica Stereoscan 440 Microscope (20 kV) (Leica Microsystems Cambridge Ltd., Cambridge, UK), equipped with an energy Dispersive Analytical System (EDS) from Inca Energy 200, by using AZtecEnergy EDS Software (v2.1, Oxford Instruments, Abingdon, UK, 2006).

A digital Optical Microscope (OM), HIROX (Hirox Co., Ltd., Tokyo, Japan), was employed to observe the morphology of the fractured composites in bending tests (see below). The observations were carried out at room temperature and by using KH-8700 Software (v1.40, Hirox Co., Ltd., Tokyo, Japan, 2013).

#### 2.3.3. TGA

TGA were performed using a Netzsch TG209 (Selb, Germany) apparatus, from room temperature up to 800 °C, at a heating rate of 10 °C/min under a nitrogen atmosphere (experimental error: ±0.5 wt %, ±1 °C) and by using Proteus Software (v4.0, Labcenter Electronics Ltd., Yorkshire, UK, 2000).

#### 2.3.4. Solid-State NMR Spectroscopy

NMR spectra were acquired with a 300-MHz (7.0 Tesla) Bruker Avance magnet (Bruker Bio Spin GmbH, Rheinstetten, Germany), composed of a wide-bore system and equipped with a CPMAS (Cross-Polarization Magic-Angle-Spinning) probe, working at ^29^Si and ^13^C frequencies of 59.62 and 75.47 MHz, respectively. Samples of Hemp (H) and Treated Hemp (HT) (80 ± 1 mg) were loaded into 4-mm zirconia rotors, closed with Kel-F caps and spun at a rate of 10,000 ± 1 Hz. ^13^C NMR spectra were acquired by applying a cross polarization technique and consisted of 1814 time domain points, a spectral width of 300 ppm (22,727.3 Hz), a recycle delay of 2 s, 4000 scans and 1 ms of contact time. The ^13^C CPMAS pulse sequence was conducted by using a 1H Ramp pulse to account for the non-homogeneity of the Hartmann-Hahn condition. ^29^Si NMR spectra were acquired by using a direct polarization and consisted of 2048 time domain points, a spectral width of 500 ppm (29,762 Hz), 40 s of recycle delay and 2730 scans.

Free Induction Decays (FIDs) were processed by BrukerTopSpin (v2.1, Bruker, Billerica, MA, USA, 2006) and MestreNOVA (v9.0, Nanalysis Corp., Mestrelab Research, Calgary, AB, Canada, 2014) Software. Prior to be phase and baseline correction, ^29^Si and ^13^C spectra were Fourier transformed by applying a two- and four-fold zero-filling and adopting an exponential filter function with a line broadening of 350 and 50 Hz, respectively.

#### 2.3.5. Cone Calorimetry Tests

To investigate the combustion behavior of the aforementioned materials subjected to a constant heat flux, cone calorimeter tests (Fire Testing Technology, East Grinstead, London, UK) were performed following the ISO 5660 standard, by using squared samples (5.0 × 5.0 × 0.3 cm^3^), with a heat flux of 35 kW/m^2^, in horizontal configuration. Time To Ignition (TTI, s), Total Heat Release (THR, MJ/m^2^), peak of the Heat Release Rate (pkHRR, kW/m^2^) were measured. Total Smoke Release (TSR, m^2^/m^2^), carbon monoxide yield (CO yield, kg/kg), carbon dioxide yield (CO_2_ yield, kg/kg) and Specific Extinction Area (SEA, m^2^/kg) were evaluated, as well. For each sample, the experiments were repeated at least three times in order to ensure reproducible and significant data.

#### 2.3.6. Three-Point Bending Tests

Three-point bending tests were carried out in an MTS (MTS Systems Corporation, Eden Prairie, MN, USA) Alliance RT/50 universal testing machine in stroke control, setting the crosshead speed at 1 mm/min in accordance with the ASTM 790M standard and by using MTS TestWorks Software (v4, MTS Systems Corporation, Eden Prairie, MN, USA, 2006). The tests were performed on specimens of 60 × 25 × 3 mm^3^; the sample span-to-depth ratio was 16:1.

#### 2.3.7. Washing Fastness

The washing fastness of the treated hemp fabrics was carried out with a solution containing 4 g/L commercial detergent; the liquor ratio was 50:1. Each wash was performed at 40 °C for 5 min. After each washing cycle, the fabric was removed, gently squeezed and rinsed with tap water. Then, repeated washing was carried out until a total of 250 min was reached.

## 3. Results and Discussion

### 3.1. Characterization of the Silica Coating

**Figure 1:**
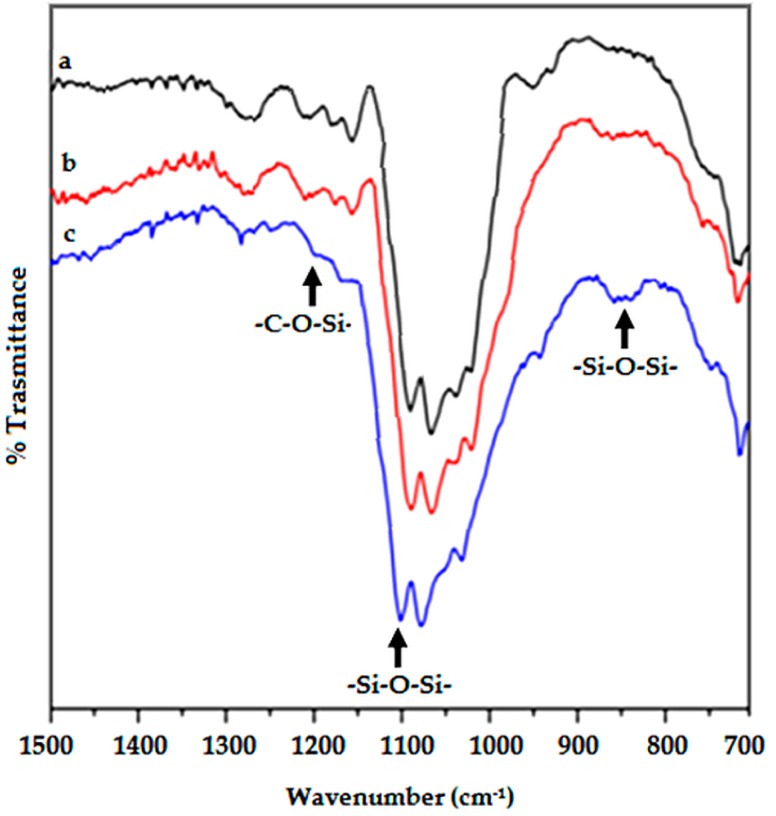
Fourier Transform Infrared (FTIR) spectra of (**a**) untreated (black line); (**b**) after two (red) and (**c**) five (blue) soaking/drying cycles.

Figure 1 shows the typical FTIR spectra of hemp fabrics before and after the treatment (two and five soaking/drying cycles) with the acidic waterglass solution. First of all, it is noteworthy that FTIR spectra modifications are consistent with the formation of a silica-based coating. In particular, the bands around 1200 and 1135 cm^−1^ may be assigned to the stretching of the –Si–O–cellulose and –Si–O–Si– bonds, respectively [27,28].

Therefore, a silica-based coating probably anchored to the hemp substrate through the formation of covalent bonds should have been formed. This finding may be ascribed to the low pH value (pH = 2.5) of the acidified waterglass solution: indeed, it is well known that when silicates are dissolved in acidic solutions, the formation of silicic acid would be expected, according to the following equation:

Na_2_SiO_3_ + H_2_O + 2HCl = Si(OH)_4_ + 2Na^+^ + 2Cl^−^

However, as soon as the reaction proceeds, the molecular units become larger, slowly giving rise to thickening phenomena that finally lead to the formation of a gel [29,30,31]. This behavior was explained by Iler [32] through a polymerization mechanism according to the following steps:

(1) formation of the particles from the precursors;

(2) subsequent growth of the particles;

(3) creation of links among the particles, which give rise to chains and networks extending throughout the liquid medium.

Harris and coworkers [33] hypothesized the formation of very small silica nanoparticles, comprising 3–7 silicon atoms linked through –Si–O–Si– siloxane bonds: this hypothesis was confirmed through NMR spectroscopy in the case of potassium silicate solutions with the K:Si atomic ratio equal to one.

The pH = 2.5 of the acidified waterglass solution, which is slightly higher than the silica isoelectric point (2.0–2.5), but lower than that reported for hemp (>3.0) [30,34,35], is expected to ensure that the silica and hemp surfaces are negatively and positively charged, respectively. This should: (i) favor silica nanoparticles to approach the fiber surface; and (ii) as long as the condensation reactions have a nucleophilic substitution mechanism, make the condensation reaction at the hemp surface preferential with respect to the reaction between silanol groups (Si–OH) in the solution.

Therefore, the formation of a silica-based coating anchored to the hemp surface through covalent bonds is reasonably supported. A mass increase of 5 wt % was observed after the hemp fabric treatment described in the Materials and Methods section. 

In order to further confirm this hypothesis, washing fastness tests have been performed according to the procedure described elsewhere [36]: the silica coating turned out to be resistant to the washing process and to be insoluble in the washing medium.

**Figure 2:**
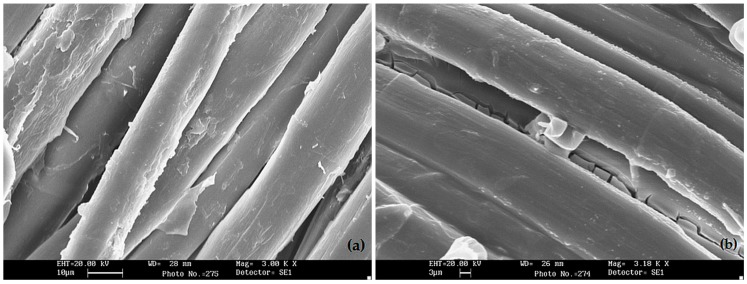
Scanning Electron Microscope (SEM) images of: (**a**) untreated hemp fabric (scale bar: 10 μm); (**b**) hemp fabric after the waterglass treatment (scale bar: 3 μm).

Figure 2 shows some typical SEM pictures of the hemp fabrics, before and after the treatment with waterglass in acidic condition: it is noteworthy that, after the formation of the waterglass coating, the surface of the fibers becomes smoother.

### 3.2. Solid-State NMR Spectroscopy

**Figure 3:**
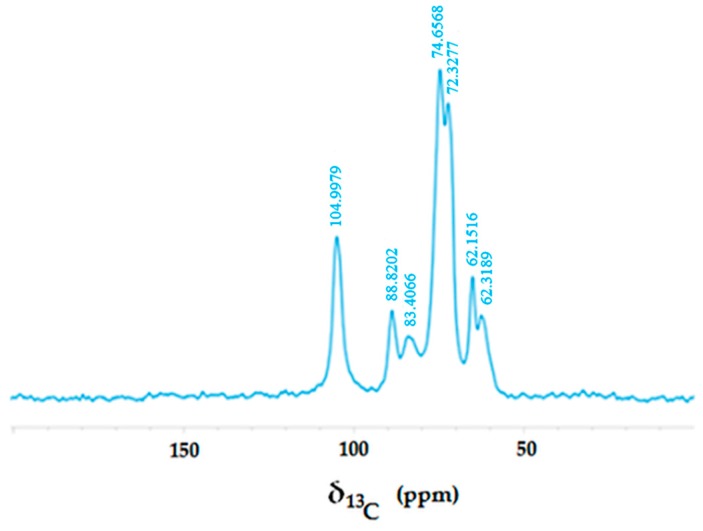
Solid-state Nuclear Magnetic Resonance (NMR) spectrum of untreated hemp fabric.

Hemp is commonly composed of cellulose, hemicellulose and lignin. The ^13^C spectrum of the H sample (Figure 3) showed no evidence of lignin, since no signal was detected in the aromatic-C spectral region.

Conversely, oligo- and poly-saccharidic compounds deriving from cellulose and hemicellulose biopolymers were revealed by the intense peaks resonating in the spectral region 54–114 ppm. In particular, the two peaks at 62.45 and 65.07 ppm were ascribed to methylene carbons belonging to different saccharidic structures. The relatively up-field resonances of these carbons were ascribed to the closeness to oxygen nuclei in the carbohydrate molecule. The peaks at 88.81 and 104.85 ppm were attributed to anomeric carbons, either in α or β forms. Their relatively down-field frequencies were explained by the scalar bonding of anomeric carbons to two deshielding oxygen atoms exerting an electron-withdrawing effect. Finally, the peaks resonating in the range 67.54–86.16 ppm were assigned to the remaining hydroxy-alkyl carbons composing oligo- and poly-saccharidic structures.

**Figure 4:**
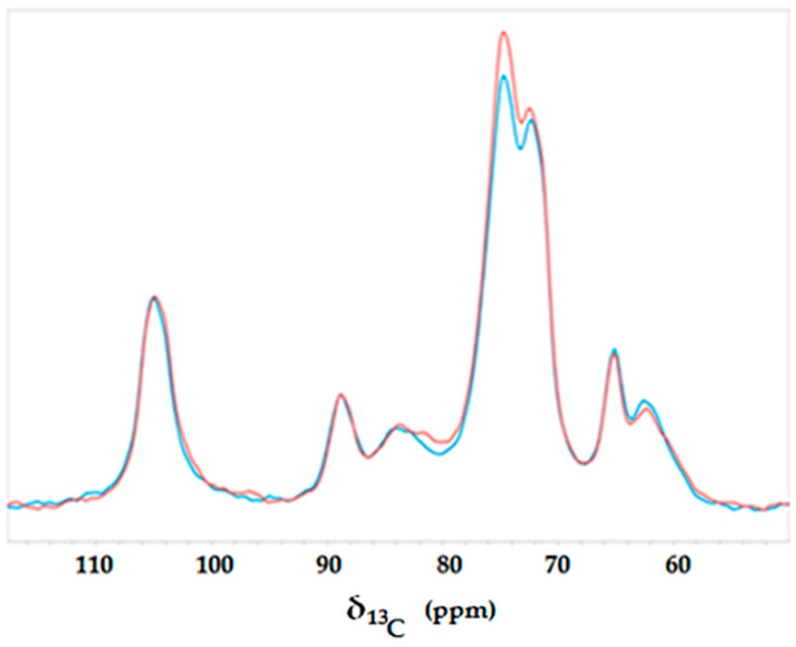
Solid-state NMR spectra of untreated hemp fabric (blue) and hemp fabric after the waterglass treatment (red).

The superimposition of the H and HT ^13^C NMR spectra is shown in Figure 4, revealing that all above-mentioned carbohydrate signals were identified also in the HT sample. However, significant differences between the two carbon spectra are evidenced. In particular, the HT sample showed several shoulders rising slightly up-field and resonating at 104.1, 81.43 and 59.83 ppm, respectively. Moreover, the peaks ranging within 67 and 78 ppm resulted in being more intense in the HT sample than in the H sample, whereas the peak at 62.15 ppm decreased in intensity and was accompanied by a slightly up-field shoulder at 59.9 ppm. These results suggest that part of the treated hemp material reacted with the applied silicate reagent. In fact, the appearance of these newly-formed resonances may be attributed to the formation of –C–O–Si– covalent bonds, such an up-field shift being due to the presence of strong electron-releasing silicon nuclei.

**Figure 5:**
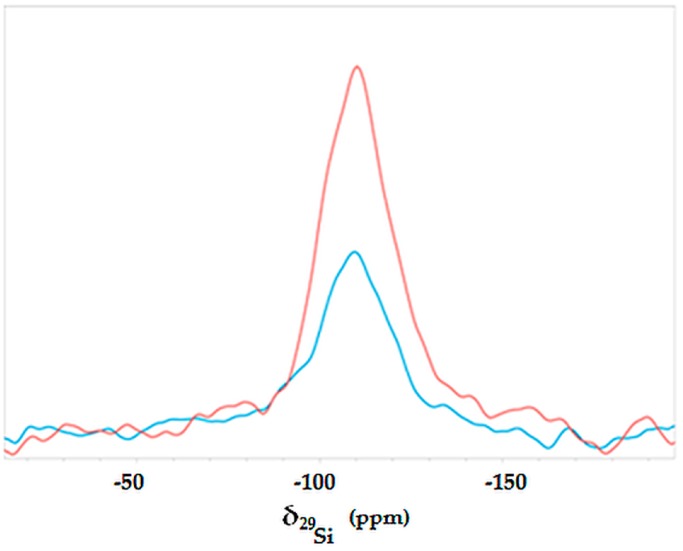
^29^Si NMR spectra of hemp (H, blue) and treated hemp (HT, red) samples acquired at a spin rate 10,000 Hz.

The superimposition of the ^29^Si spectra of H and HT samples is shown in Figure 5. It is well known, in fact, that plants do uptake from the soil solution some soluble silicates and salts, aiming at strengthening the robustness of their aerial parts. In plants, silica can be up to 10% of the total plant weight [37]. When comparing the curves of Figure 5, significant changes are appreciated as a consequence of the surface treatment. In both samples, two intense and broadened signals were detected at 93.04 and 109.6 ppm and attributed to silicon nuclei forming polysiloxane chains in Q3 and Q4 forms, respectively [38]. Despite Q3 and Q4 Si nuclei being detected in both the H and HT samples, the intensity of both peaks was significantly higher in the case of the reaction product. In addition, the finding that each of these peaks appeared relatively broadened is indicative of the coexistence of amorphous and crystalline forms of the polysiloxane network. As shown in Figure 5, the H sample exhibited an intense signal centered at 92 ppm, thus suggesting a relatively large abundance of Q3 Si, which is presumably bound to a single hydroxyl. This resonance almost totally disappeared in the HT sample, whereas a pronounced shoulder emerged up-field at 102.97 ppm. In line with the findings described for carbon spectra, this change can be due to the presence of Q3 Si nuclei, whereby the Si–OH group reacted to form a –C–O–Si– bond.

### 3.3. Thermogravimetric Analysis

**Figure 6:**
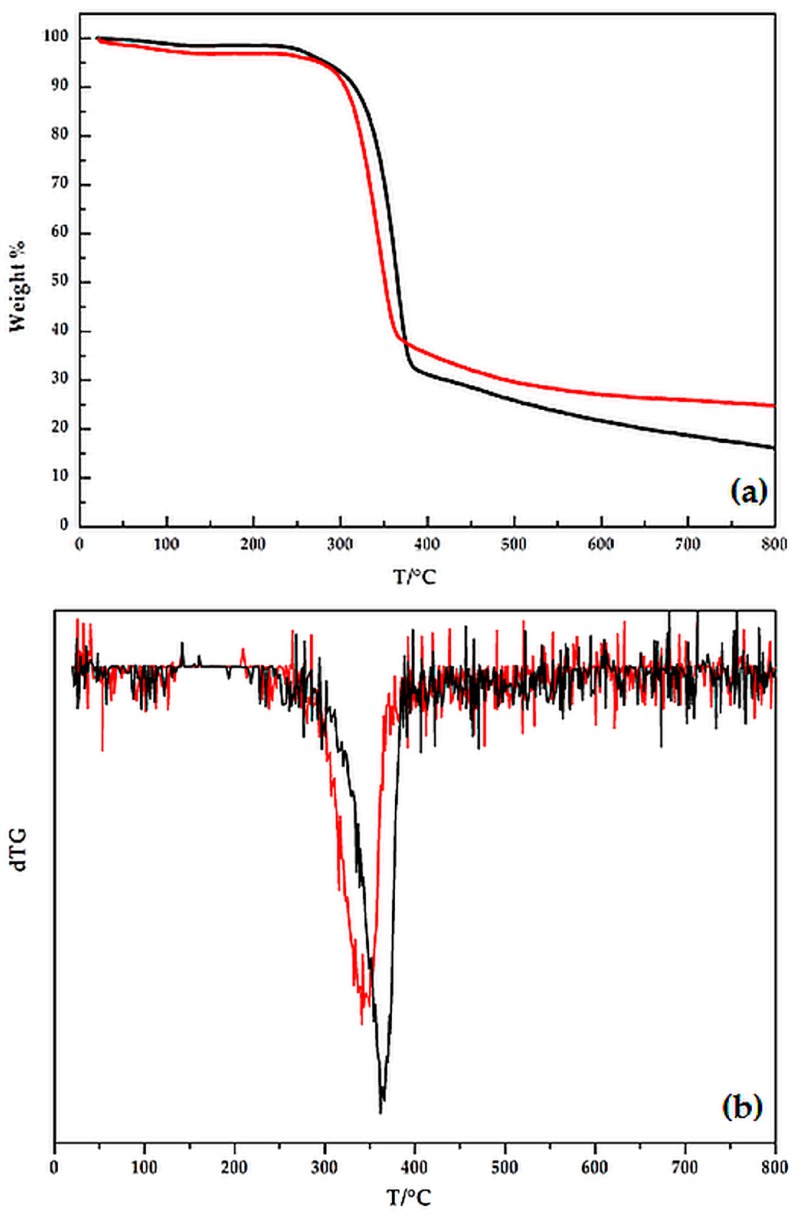
Thermogravimetry (TG) (**a**) and dTG (**b**) curves in an inert atmosphere for hemp, before (black line) and after (red line) the waterglass treatment.

Figure 6 shows the thermogravimetry (TG) curves in an inert atmosphere of hemp, before and after the treatment with waterglass in acidic conditions. Table 2 collects the corresponding TG data: T_5%_, T_10%_ and T_50%_ are the temperatures at which 5%, 10% and 50% weight loss are recorded; the residues at 800 °C and at the temperature at which the weight loss rate reaches the maximum are also reported. The thermal behavior of hemp can be interpreted on the basis of the scheme of the thermal degradation mechanism reported in the literature [39], which involves two stages:

(1) Stage I (between 300 and 400 °C): this involves two competing pathways that yield aliphatic char and volatiles.

(2) Stage II (between 400 and 800 °C): some of the aliphatic char converts into an aromatic form.

According to the reported scheme, the TG curves recorded in an inert atmosphere (Figure 6) show one main degradation step in the temperature range 300–400 °C; furthermore, a slight mass loss is observed in between 80 and 150 °C for both fabrics and could be ascribed to humidity loss.

It is worthy to note that the waterglass treatment, because of the acidic characteristics of the deposited coating, anticipates the thermal degradation of the cellulosic fibers (see the T_5%_ and T_10%_ values of Table 2), but at higher temperatures exerts a protective effect on the substrate, significantly increasing the residue at high temperatures (30.4% vs. 22.1%, for waterglass-treated and pristine hemp, respectively).

**Figure 7:**
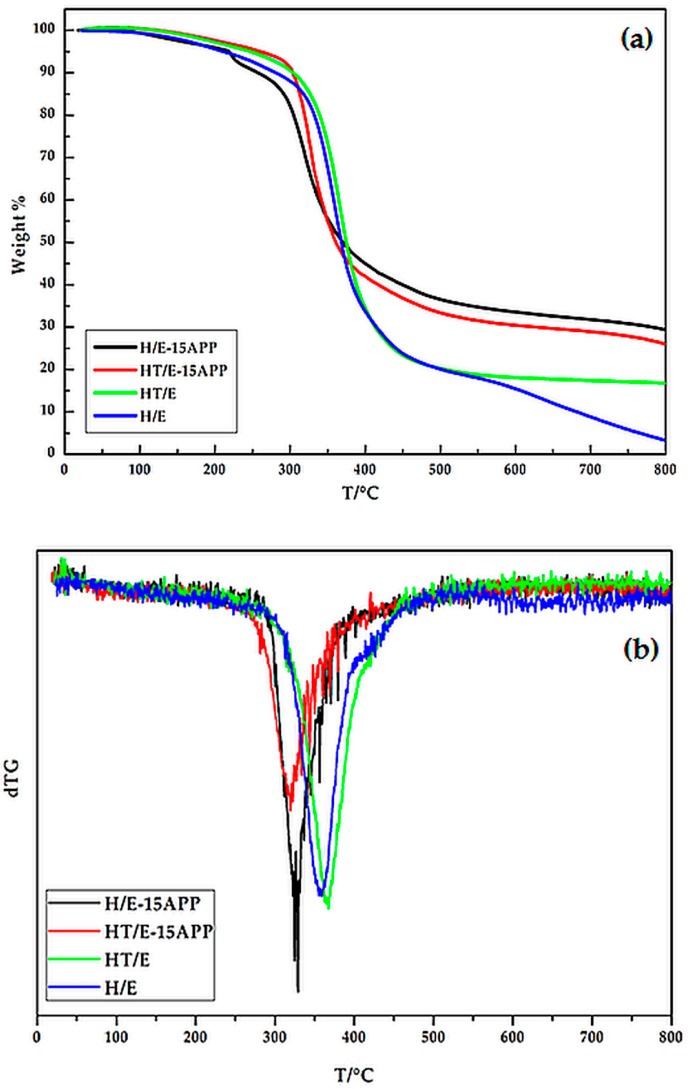
TG (**a**) and dTG (**b**) curves in an inert atmosphere for the investigated composite.

When hemp is embedded in the epoxy resin, the waterglass treatment increases the overall thermal stability of the composites, giving rise to a significant increase of the residues at high temperatures (23**%** vs. 9.9**%** for HT/E and H/E composites, respectively; Table 2). The TG and dTG curves of the composites are plotted in Figure 7. As far as the composites reinforced with untreated hemp fabrics (i.e., H/E-15APP) are concerned, the presence of 15% of APP in the epoxy matrix determines an anticipation of the degradation phenomena, with respect to H/E counterparts, notwithstanding a significant increase of the final residue at high temperatures. This behavior could be ascribed to the presence of the flame retardant additive, as already reported in the literature [40]. Conversely, the waterglass treatment in combination with the presence of APP turns out to remarkably increase the thermal stability of the obtained composites (compare the last two rows of Table 2): this finding could be ascribed to a joint effect occurring between waterglass and APP during the heating up of the composite material.

### 3.4. Cone Calorimetry Tests

Table 3 and Table 4 collect the cone calorimetry data: TTI (s); time to Flame Out (s) (FO); (HRR) (average) (kW/m^2^); pkHRR (kW/m^2^); THR (MJ/m^2^); TSR (m^2^/m^2^); mass residue at the end of the cone calorimeter test; SEA(m^2^/kg); carbon monoxide and dioxide yields (kg/kg).The first two lines refer to untreated (H) and waterglass-treated (HT) fabrics. It is worthy to note that the waterglass coating, despite an anticipation of the ignition of the fabric, is responsible for a slight decrease of HRR, pkHRR and THR and for a limited increase of the final residue, as well, hence further showing its protective effect exerted on the underlying fabric.

Conversely, the hemp fabric surface treatment seems to be very effective in improving the fire behavior of the prepared composites. Indeed, the presence of the waterglass coating significantly reduces the HRR by 35%, namely from 402 down to 260 kW/m^2^ (see the values for H/E and HT/E, respectively). A similar trend is observed as far as pkHRR is considered: its decrease is as much as −14.9% (from 754 down to 642 kW/m^2^, for H/E and HT/E, respectively). On the other hand, THR does not seem to be affected by the treatment of the fabrics with the waterglass coating, while the latter shows a detrimental effect on TTI, which is reduced from 55 down to 39 s.

A significant role is played by APP: indeed, this flame retardant additive, despite a reduction of TTI, favors a further remarkable decrease of HRR (−77.6% and −73.8%, for H/E-15APP and HT/E-15APP, respectively), pkHRR (−65.6% and −63.8%, for H/E-15APP and HT/E-15APP, respectively) and THR (−43.9% and −37.5%, for H/E-15APP and HT/E-15APP, respectively), with respect to the unfilled composite counterparts (i.e., H/E and HT/E samples).

In addition, the waterglass coating, also in combination with APP, seems to limit the smoke formation of the epoxy composites: in particular, APP turns out to significantly decrease both TSR and SEA parameters. It is noteworthy that the phosphorus additive seems to be more effective when added to the composites where hemp fabrics have not been subjected to the treatment with waterglass. As an example, TSR is decreased by 58.4% and SEA by 46.4%, as well, when 15% of APP is added to the composite (compare the H/E and H/E-15APP samples).

**Figure 8:**
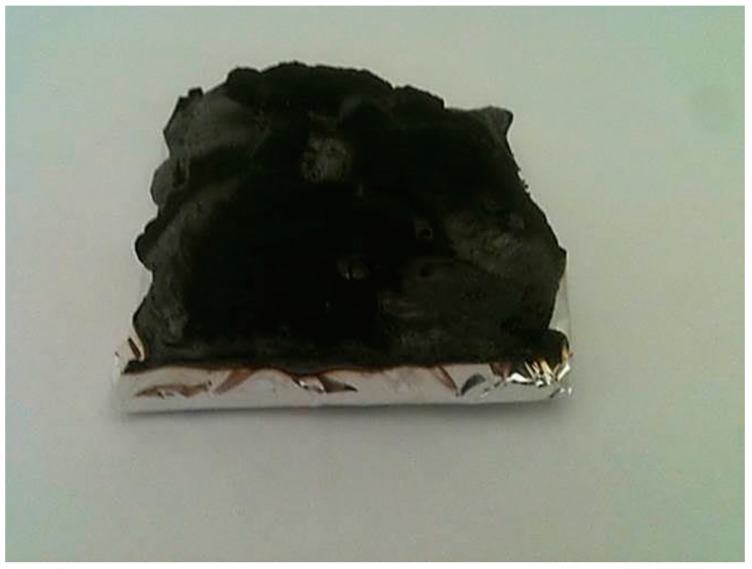
Residue of H/E-15APP after cone calorimetry tests.

**Figure 9:**
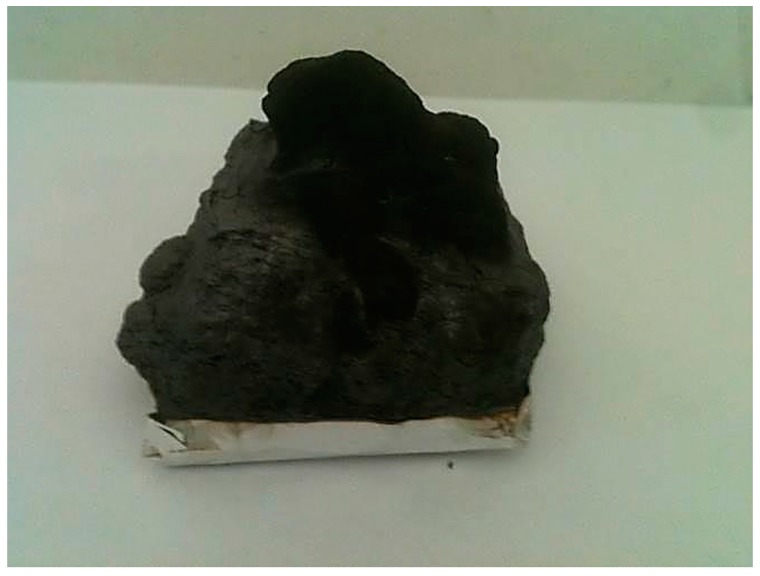
Residue of HT/E-15APP after cone calorimetry tests.

The very high residues after cone calorimetry tests for H/E-15APP and HT/E-15APP (some pictures are shown in Figure 8 and Figure 9) seem to indicate that the acidic character of the waterglass coating, in combination with the presence of APP, could favor the dehydration reactions of the fabric and of the epoxy resin, hence giving rise to the formation of a very stable char.

### 3.5. Three-Point Bending Tests

The hydrophilic behavior of natural fibers has a low compatibility with the hydrophobic polymer matrix; in addition, waxes and other non-cellulosic substances, which determine poor adhesion between matrix and fibers, cover the surface of the latter, as pointed out in the scientific literature [41,42,43,44].

**Figure 10:**
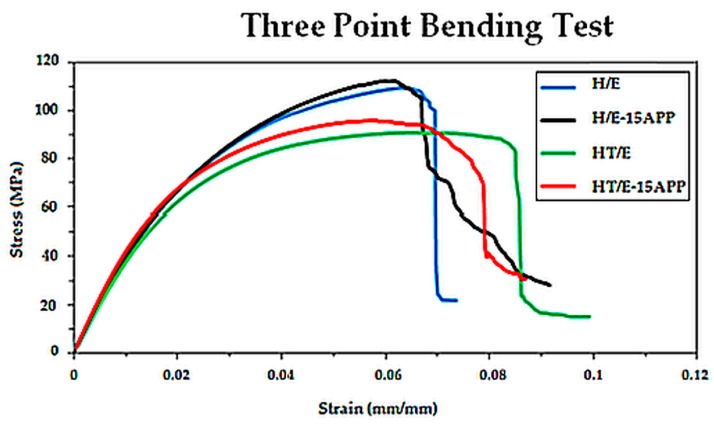
Stress-strain curves for the four types of composites: H/E sample, H/E-15APP sample, HT/E sample, HT/E-15APP sample.

In order to evaluate the effect of the presence of APP in the epoxy matrix and of the waterglass treatment of the hemp fabrics on the mechanical behavior of the obtained composites, three-point bending tests were carried out in accordance with the ASTM 790M standard. Figure 10 plots the stress-strain curves for the different laminates. Table 5 collects the average values of the flexural modulus, flexural strength and maximum strain for the different laminates.

**Figure 11:**
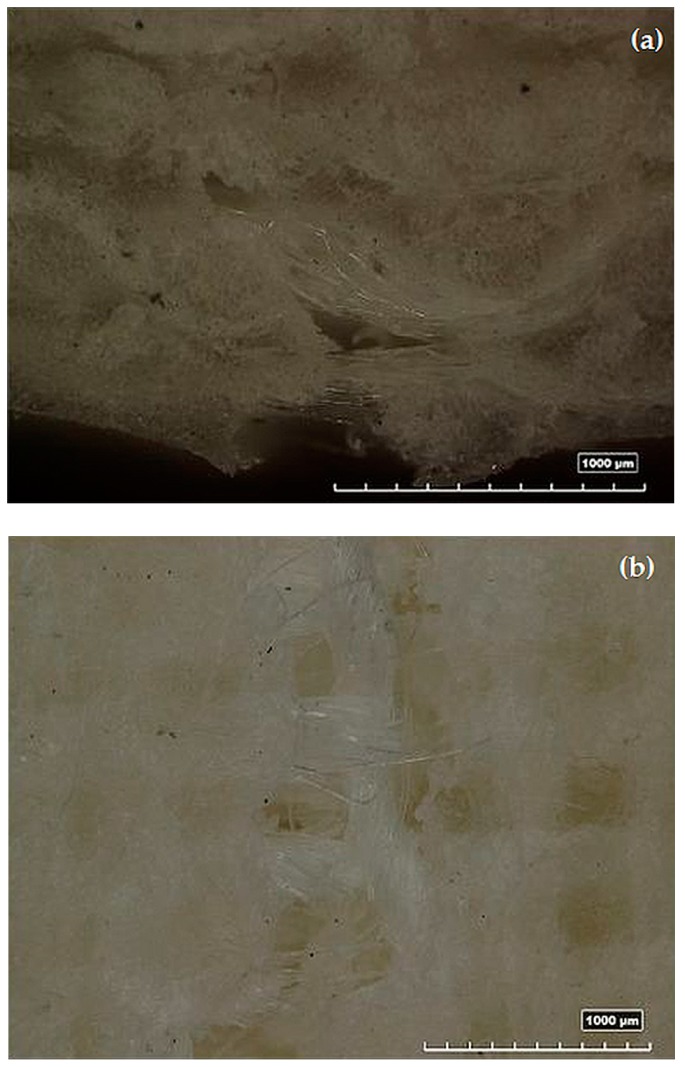
Images of the fracture for the tensile stress in the section (**a**) (140×) and in the plane (**b**) (100×) view.

For all of the tested specimens, an evident pullout, showing a poor fiber/matrix adhesion, characterized the breakage for tensile stress. The photographs of the fracture on the tensile site, in section (140×) and in plane (100×) view, are shown in Figure 11a,b, respectively.

As shown in Figure 10, all of the types of composites show the same initial slope, but, as the strain increases, the behavior is different, according to the treatment the fabrics have been subjected to: in particular, the waterglass treatment of the fibers could give rise to a worse adhesion at the interface fiber-matrix, hence decreasing the maximum stress and increasing of the elongation at break.

Furthermore, the presence of APP in the epoxy matrix does not seem to affect the overall mechanical behavior of the obtained composites.

## 4. Conclusions

In this paper, hemp fabrics have been treated with a new, inexpensive, simple and environmentally-friendly silica-based coating, able to protect the fabrics from heat sources, hence improving their fire behavior when utilized as reinforcing agents in epoxy-based composites. To the best knowledge of the authors, this is the first time that inexpensive waterglass solutions were exploited for this purpose: indeed, the proposed approach can be considered as an application of sol-gel chemistry using a precursor that does not need hydrolysis and is performed in water solutions, avoiding the organic solvents usually required by the alkoxy precursors. FTIR and solid-state NMR spectroscopies have confirmed the formation of –C–O–Si– covalent bonds in between the waterglass silica coating and the underlying fabrics.

The waterglass treatment, which is resistant to washing, seems to be very effective at improving the fire behavior of hemp fabric/epoxy composites, also in combination with ammonium polyphosphate. In particular, the concurrent presence of hemp surface treatment and APP significantly improves such cone parameters as HRR, THR, TSR and SEA, which turn out to decrease respectively by 83%, 35%, 45% and 44% as compared to untreated hemp/epoxy composites. At the same time, the formation of a very stable char is promoted, as also assessed by TG analysis performed in an inert atmosphere.

Conversely, the low interfacial adhesion between the fibers and the epoxy matrix promotes a brittle behavior of the composites, which show a slightly lower stiffness as compared to the theoretical one. However, the fracture energy absorbed by the material reinforced by the waterglass-treated silica coating is higher. Finally, the presence of APP in the epoxy matrix does not seem to affect the mechanical behavior of the obtained composites.

## Figures and Tables

**Table 1:** Samples investigated. APP, Ammonium Polyphosphate; H, untreated hemp fabrics; HT, waterglass-treated hemp fabrics.

Symbol	Hemp fabric and epoxy composite samples	Amount of APP added to the epoxy (wt %)
H	Hemp fabrics	0
HT	Hemp fabrics treated with waterglass	0
H/E	Hemp fabrics/epoxy composite	0
HT/E	Hemp fabrics treated with waterglass/epoxy composite	0
H/E-15APP	Hemp fabrics/epoxy composite	15
HT/E-15APP	Hemp fabrics treated with waterglass/epoxy composite	15

**Table 2:** Thermogravimetry (TG) data obtained in an inert atmosphere.

Sample	T_5%_ (°C)	T_10%_ (°C)	T_50%_ (°C)	T_peak_ (°C)	Residue at T_peak_ (%)	Residue at 800 °C (%)
H	274	296	338	367	36	22
HT	264	283	324	342	43	30
H/E	221	274	342	357	41	9.9
HT/E	248	289	349	357	40	23
H/E-15APP	203	251	364	328	57	35
HT/E-15APP	264	383	344	319	60	32

**Table 3:** Results from cone calorimetry tests performed on hemp and on the different composites before and after the waterglass treatment. TTI, Time To Ignition; FO, time to Flame Out; HRR, Heat Release Rate; pkHRR, peak of the Heat Release Rate; THR, Total Heat Released.

Sample	TTI (s)	FO (s)	HRR (kW/m^2^)	PkHRR (kW/m^2^)	THR (MJ/m^2^)	Residue mass (%)
H	28 ± 7.0	56 ± 2.5	13.2 ± 0.951	57.8 ± 9.77	1.80 ± 0.264	1 ± 0.6
HT	21 ± 2.6	45 ± 4.5	12.3 ± 1.48	51.8 ± 10.4	1.60 ± 0.252	4 ± 0.6
E	78 ± 6.9	166 ± 14.2	507 ± 120	1937 ± 119.3	95.8 ± 8.03	3 ± 0.6
H/E	55 ± 4.0	178 ± 11.9	402 ± 22.6	754 ± 85.7	61.3 ± 1.73	3 ± 0.6
HT/E	39 ± 4.0	187 ± 6.42	260 ± 13.3	642 ± 72.6	64.2 ± 4.65	6.71 ± 0.577
H/E-15APP	46 ± 8.1	336 ± 49.1	90 ± 12	259 ± 16.5	34.4 ± 1.47	28.7 ± 0.577
HT/E-15APP	44 ± 4.2	557 ± 97.1	68 ± 21	232 ± 45.1	40.1 ± 7.59	30.3 ± 0.577

**Table 4:** Smoke results from cone calorimetry tests performed on hemp and on the different composites before and after the waterglass treatment. TSR, total smoke release; SEA, specific extinction area; ND, not detectable.

Sample	TSR (m^2^/m^2^)	SEA (m^2^/kg)	CO yield (kg/kg)	CO_2_ yield (kg/kg)
H	ND	ND	ND	ND
HT	ND	ND	ND	ND
E	3,276 ± 449	849 ± 55.4	5.85 × 10^−2^ ± 4.91 × 10^−3^	1.96 ± 4.27 × 10^−2^
H/E	2,254 ± 77.3	735 ± 22.7	3.28 × 10^−2^ ± 1.08 × 10^−3^	1.69 ± 1.01 × 10^−2^
HT/E	2,094 ± 229	667 ± 43.7	4 × 10^−2^ ± 1 × 10^−3^	1.47 ± 7.37 × 10^−2^
H/E-15APP	938 ± 68.2	394 ± 33.3	5 × 10^−2^ ± 3 × 10^−3^	0.87 ± 2.6 × 10^−1^
HT/E-15APP	1,230 ± 52.6	413 ± 16.3	5 × 10^−2^ ± 2 × 10^−3^	1.02 ± 1.94 × 10^−1^

**Table 5:** Results from the three-point bending tests.

Sample	Flexural modulus (MPa)	Flexural strength (MPa)	Maximum strain (%)
H/E	4,550 ± 260.1	109 ± 5.61	7.1 ± 6.7 × 10^−1^
H/E-15APP	4,590 ± 324.3	110 ± 6.12	6.9 ± 7.7 × 10^−1^
HT/E	4,340 ± 194.1	92 ± 4.8	8.5 ± 8.8 × 10^−1^
HT/E-15APP	4,460 ± 258.2	94 ± 5.2	8.2 ± 9.1 × 10^−1^
